# The therapeutic potential of Ma'aljobon, a whey product, in primary hypertension: A double-blind randomized controlled trial

**DOI:** 10.22038/AJP.2024.24921

**Published:** 2025

**Affiliations:** 1 *Department of Traditional Pharmacy, Institute for Studies in Medical History, Persian and Complementary Medicine, School of Persian Medicine, Iran University of Medical Sciences, Tehran, Iran *; 2 *Department of Traditional Medicine, Institute for Studies in Medical History, Persian and Complementary Medicine, School of Persian Medicine, Iran University of Medical Sciences, Tehran, Iran*; 3 *Department of Persian Medicine, Birjand University of Medical Sciences, Birjand, Iran*; 4 *Cardiovascular Diseases Research Center, Birjand University of Medical Sciences, Birjand, Iran*; 5 *African Medicines Innovations and Technologies Development (AMITD), Indigenous knowledge based Medicines and Innovations Centre, School of Clinical Medicine, Faculty of Health Sciences, University of the Free State, Bloemfontein 9300, South Africa*; 6 *Department of Pharmacology, African Medicines Innovations and Technologies Development (AMITD), Indigenous knowledge based Medicines and Innovations Centre, School of Clinical Medicine, Faculty of Health Sciences, University of the Free State, Bloemfontein 9300, South Africa*

**Keywords:** Hypertension, Persian medicine, Whey proteins, Traditional medicine

## Abstract

**Objective:**

Ma’aljobon is used in Persian medicine (PM) as a natural antihypertensive product. This study aimed to evaluate the hypotensive effect of Ma’aljobon in patients with uncontrolled grade 1 primary hypertension (HTN).

**Materials and Methods:**

This double-blind, placebo-controlled clinical trial included 114 patients (20-80 years) with uncontrolled grade 1 primary HTN. After obtaining informed consent, the participants were randomly divided into two groups and administered with 25g of Ma’aljobon or maltodextrin twice daily for six weeks. Systolic and diastolic blood pressures (SBP and DBP, respectively) were analyzed.

**Results:**

A total of 97 patients (52.3±10.7 years, %53.6 female) completed the study. In the Ma’aljobon group, SBP decreased from 150.3±12.3 to 130.6±12.1 mm Hg, and DBP decreased from 93.3±8.2 to 80.1±6.6 mm Hg (p<0.001). In the control group, SBP decreased from 147.6±11.2 to 138.7±14.4 mm Hg, and DBP decreased from 86.6±7.7 to 82.2±8.2 mm Hg (p<0.001). There was a significant difference in the changes of SBP and DBP between the two groups over time (p<0.001). No adverse events were observed.

**Conclusion:**

Ma’aljobon has a stronger hypotensive effect than placebo in patients with HTN and can be recommended as an add-on therapy for uncontrolled HTN.

## Introduction

Hypertension (HTN) stands as a primary, modifiable risk factor for cardiovascular disease and overall mortality globally. HTN is a major global health concern with an estimated 1.56 billion patients expected to face it by 2025 (Yang, 2017; Mills et al., 2020). Despite widespread utilization of antihypertensive therapies over the past four decades, global average blood pressure levels have either stabilized or modestly declined. Conversely, the proportion of individuals diagnosed with HTN has surged, particularly in low- and middle-income nations (Mills et al., 2020). 

Persian medicine (PM) is one of the oldest medical schools in the world, with roots in knowledge, wisdom, and experiential learning (Pasalar, 2021). In PM, HTN is believed to be analogous to “*Imtila”* which refers to the fullness of the body's ducts and spaces with improper and raw materials (Ghods et al., 2014). One of the six essential principles of PM for maintaining health is achieving a balance between what we eat and what we excrete (Siahpoosh, 2012). If an imbalance occurs between them, waste products may accumulate in the body and cause excessive fullness of blood vessels with improper materials. This condition, known as “*Imtila*” in Persian medical texts, can impair organ and cell function and lead to disease (Ghods et al., 2014). 

The relationship between BP and nutrition factors, specifically amino acids and organic acids, has gained significant interest in recent times (Hou et al., 2017; Miura and Torii, 2012; Tian and Liang, 2021). Amino acids have a direct effect on kidney function, resulting in an increase in renal plasma flow, glomerular filtration rate, and sodium excretion, thereby affecting BP levels (Rebholz et al., 2012; Tian and Liang, 2021).

Ma’aljobon as a functional food closely resembled that of a Whey protein (WP) and therefore, would host possible bioactive amino acids as seen with WP (Madureira et al., 2007). WPs are widely acknowledged, with longstanding recognition for their superior nutritional quality, characterized by high digestibility and bioavailability (Mathai et al., 2017). Notably, WP incorporates a range of biologically active constituents, including bioactive peptides and essential amino acids. These components such as the amino acids, tryptophan, arginine, and tyrosine can hold promise for functionality in a range of biological functions and the promotion of human health (Hou et al., 2015). Essential amino acids may exert an influence on a large spectrum of biological functions such as changes in metabolism, immune regulation, and especially, cardio-vascular function (Madureira et al., 2010; Madureira et al., 2007). A meta-analysis of more than 3000 participants demonstrated a direct correlation between decreases in SBP and DBP with increases in protein intake when compared to carbohydrate intake alone (Rebholz et al., 2012). 

Ma’aljobon is produced through various methods in PM literature. It is a liquid obtained during cheese production by adding rennet or acid (such as vinegar or lemon juice). The whey produced varies in mineral composition, acidity levels, and protein ratio depending on the method used. The quality of whey produced through different methods is assessed based on the levels of its primary proteins, alpha-lactalbumin (ALA) and beta-lactoglobulin (BLG). In this research, the acidotic method was selected to achieve the highest percentage of ALA and BLG contents to enhance its efficacy in treating HTN (Hosseini et al., 2017). ALA is rich in branched-chain amino acids (BCAAs) and is recognized as a globular protein that contains various amino acids, including tryptophan and cysteine (Sandström et al., 2008). Additionally, due to its similarity to human milk composition, ALA is commonly used in commercial formulations for infant supplementation (Lönnerdal, 2014). BLG serves as a source of essential branched-chain amino acids and is a noncovalent homodimer found in cow milk; it is not present in human milk as it belongs to the lipocalin family class (Brownlow et al., 1997). Intriguingly, molecular studies on BLG have revealed the presence of tryptophan amino acid sequences in it using fluorescence studies and molecular modeling techniques (Barbiroli et al., 2022; Geng Sheng et al., 2020). Research has shown that chronic tryptophan treatment led to improved renal function after HTN induction in salt-sensitive rats (Yang et al., 2022). Moreover, studies have shown that the sustained dietary provision of the neutral amino acid L-tryptophan can mitigate the progression of hypertension induced by deoxycorticosterone acetate (DOCA), renal dysfunction, and spontaneous genetic factors (in the SHR rat model), without significant alterations in their food consumption or body mass (Cade et al., 1990).

Based on the properties outlined for Ma'aljobon and our prior studies (Navabzadeh et al., 2019a; Navabzadeh et al., 2019b), this study aims to evaluate its hypotensive effect in patients with uncontrolled grade 1 primary HTN, considering the lack of evidence for its hypotensive effect.

## Materials and Methods

### Trial design

This randomized, placebo-controlled, double-blind clinical trial was performed from fall 2017 to spring 2018 at a cardiology clinic in Birjand City, South Khorasan Province, Iran. The study protocol was registered on the Iranian Registry of Clinical Trials website (No. IRCT20140519017756N34). 

### Participants

The study included patients with grade 1 HTN whose BP was not controlled despite receiving standard treatments and was still categorized as grade 1 primary HTN as per 2018 European Society of Hypertension guidelines (SBP= 140-159 mmHg, and DBP= 90-99 mmHg) (Williams et al., 2018), aged 20-80 years, with body mass index (BMI) <30. To reduce the influence of standard antihypertensive medications on study outcomes, only those who had not changed their medication or did not receive dosage adjustment for at least a month, entered the study. This approach ensures that the study results were not influenced by the effects of new medications. Due to the diverse range of standard antihypertensive medications used by the patients, patients were allowed to enter the study at any drug dosage or drug class. At the end of the study, medication details were recorded and analyzed to see how the intervention affected different drug classes outcomes.

The exclusion criteria were SBP≥160 mm Hg, DBP≥100 mm Hg, secondary HTN, end-organ damage, cardiac arrhythmias, symptomatic heart valve disease (except for mitral valve prolapse), diabetes, liver function impairment (cirrhosis, chronic active hepatitis, or autoimmune hepatitis), or kidney function impairment (i.e. creatinine more than 1.5 mg/dL), platelets less than 100,000, Partial Thromboplastin Time (PTT)>1.5).

After obtaining written informed consent, a calibrated mercurial sphygmomanometer was used to measure BP (Riester, Model: 0124, Germany). Each patient’s BP was checked twice from the right arm during each visit by the same investigator, and mean SBP and DBP were recorded after 10 min of rest.

### Interventions

For 6 weeks, patients in the intervention group took Ma’aljobon in addition to standard antihypertensive medications, with little to no change in their usual dietary intake. Then, 25 g of Ma’aljobon was given twice a day; at fasting time in the morning and 6:00 p.m., Ma’aljobon powder was dissolved in 200 ml of lukewarm water. After taking the drug, patients were instructed to go for a short walk and told not to sleep or take a bath for 3 hr. The control group took maltodextrin powder (made from corn starch) in the same way.

### Drug and placebo preparation

Ma’aljobon was ordered to Niak Pharmaceutical Company to be prepared, applying the best method of Ma’aljobon production, which was described by Mirabzadeh et al (Mirabzadeh et al., 2015). Maltodextrin was also requested to be included in the same package by Niak Pharmaceutical Company.

The recipe for Ma’aljobon was as follows: 1800 kg of fresh cow milk was boiled for 20 min. Once the temperature dropped to 75°C, 250 kg of oxymel and 5 kg of vinegar were added to facilitate separation into components. The resulting solution was then spray-dried at a rate of 250 liters per hour, with an inlet temperature of 120°C and an outlet temperature of 50°C. To mitigate protein degradation due to heat, the company employed a freeze-drying apparatus to dehydrate the obtained whey protein. Ultimately, each 300 g portion was packaged in polyethylene containers. Compared to other recipes, the levels of ALA and BLG in this product were higher, measuring 8093 ppm and 11635 ppm, respectively, as already reported by Mirabzadeh et al (Mirabzadeh et al., 2015). 

Maltodextrin powder served as a placebo in this trial. Maltodextrin is formed by the enzyme alpha-enzymatic amylase’s hydrolysis of cornstarch, resulting in the development of a molecule with a lower molecular weight known as dextrin or maltodextrin (Fekete et al., 2016).

### Outcomes

#### Primary outcome

 BP was measured every 2 weeks during the study period (6 weeks) by the researchers at the clinic.

#### Secondary outcomes

At baseline and the end of the sixth week, blood biochemistry tests were performed, including fasting blood sugar (FBS), cholesterol (Chol), triglyceride (TG), low-density lipoprotein (LDL), high-density lipoprotein (HDL), alanine aminotransferase (ALT), aspartate aminotransferase (AST), and hematocrit (HCT).

### Sample size

Using Cohen’s formula for the calculation of sample size (Glen.) and considering a medium effect size of 0.6 for SBP and DBP, type I error of 0.5, power of 80%, and attrition rate of 20%, 57 participants were considered per group.

$n=\frac{2{\left(Z_{\frac{a}{2}}+Z_{\beta }\right)}^{2}}{d^{2}}$

$d=\frac{\mu_{1}-\mu_{2}}{\sigma }$

**Table 1:** Demographic and baseline characteristics of the participants

**Variable**						**p** ** value**
		**Placebo(N:40) **		**Ma’aljobon(N:57) **		
Age (year)*		52.6±11.1		52.2±10.6		0.87
Sex *n* (%)	Male	11(27.5)		34(59.6)		0.002
	Female	29(72.5)		23(40.4)		
BMI (kg/m^2^)*		28.2±2.2		27.2±2.4		0.06
Marriage *n *(%)	Single	3(7.5)		6(10.5)		0.16
	Married	37(92.5)		51(89.5)		
Job *n* (%)	Employed	20(50)		38(66.6)		0.14
	Unemployed	20(50)		19(33.3)		
Smoking *n *(%)		3(7.5)		10(17.5)		0.22
OCP *n* (%)		17(42.5)		16(28.1)		0.19
Duration of HTN (months)*		61.1±6		69.6±70.1		0.54
Medication use *n* (%)		1	21(52.5)	1	42(73.7)	0.09
		2	16(40)	2	13(22.8)	
		3	3(7.5)	3	2(3.5)	
Duration of medication use (months) *		62.3±67.7		44.7±58		0.17
SBP (mm Hg)		147.6±11.2		150.3±12.3		0.002
DBP (mm Hg)		86.6±7.7		93.3±8.2		<0.001
Used antihypertensive medications						
Diuretic n (%)		11 (27.5)		6 (10.5)		0.05
Beta blocker (BB)**n (%)		20 (50)		19 (33.3)		0.09
Angiotensin-converting enzyme inhibitor- I (ACEI-I)**		0		2 (3.5)		0.51
Angiotensin-converting enzyme inhibitor-II (ACE-II)**		26 (65)		28 (49.1)		0.12
Calcium Channel Blocker (CCB)**		5 (12.5)		10 (17.5)		0.57

BMI: body mass index; SD: standard deviation; n: number; OCP: oral contraceptive pills; SBP: systolic blood pressure; DBP: diastolic blood pressure.^*^Mean ± Standard deviation,^**^ n (%).

**Figure 1:**
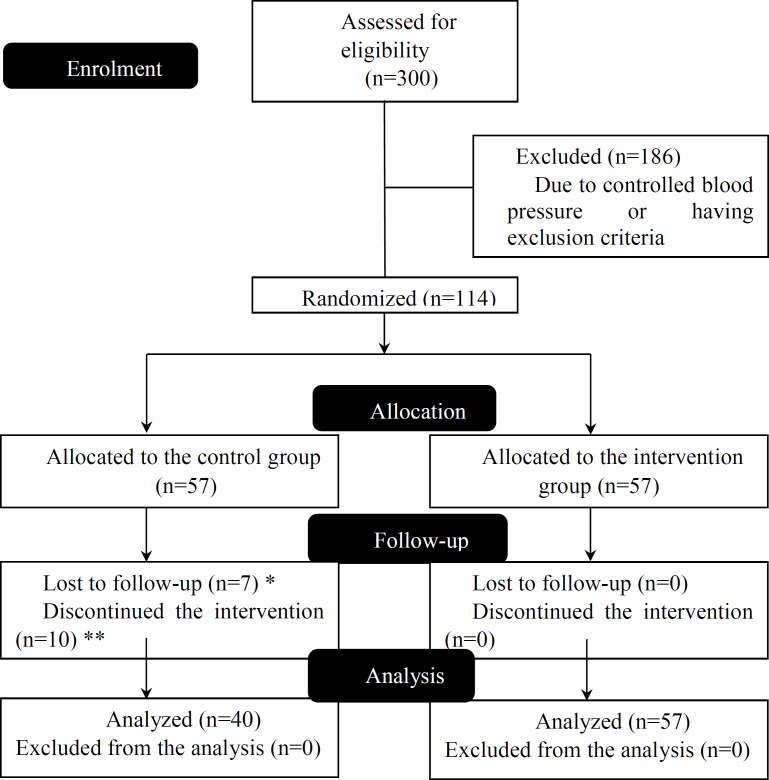
The trial flow chart. * Four people lived in rural areas, 2 suffered from severe hypertension, and one had kidney stone surgery. ** Six were not satisfied with the medication’s taste, and 4 did not answer the phone.

**Figure 2:**
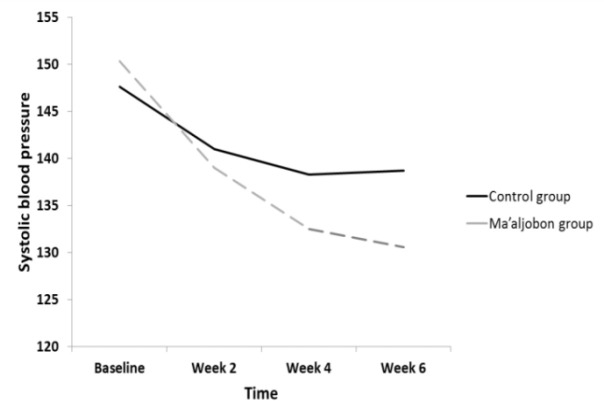
The trend of SBP in both groups from baseline to week 6

**Figure 3:**
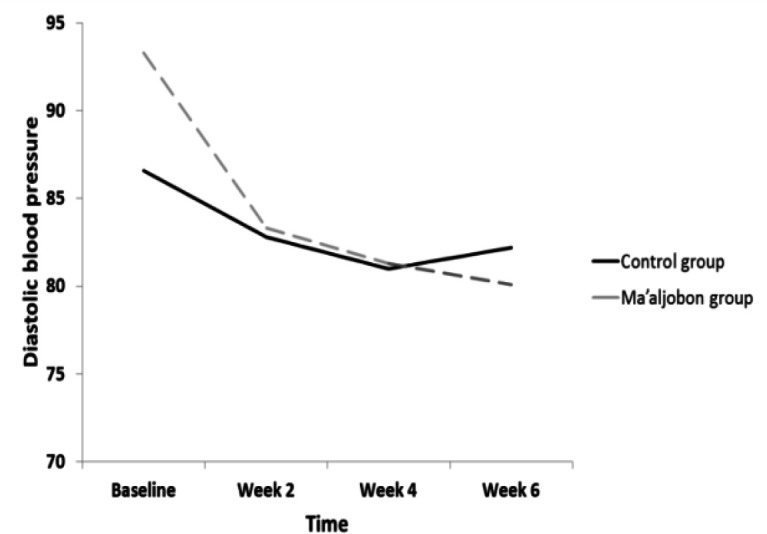
The trend of DBP in both groups from baseline to week 6

**Table 2:** SBP (mmHg) and DBP(mmHg) in each group during the study

**Time**	**SBP**			**DBP**		
	**Control group** **Mean±SD**	**Ma’aljobon group** **Mean±SD**	**p** value*	**Control group** **Mean±SD**	**Ma’aljobon group** **Mean±SD**	**p** value*
Baseline	147.6±11.2	150.3±12.3	0.002	86.6±7.7	93.3±8.2	<0.001
Week 2	141±16.1	139±13.1	0.519	82.8±7.1	83.3±6.1	0.706
Week 4	138.3±17.4	132.5±11.5	0.072	81±7.6	81.3±6	0.723
Week 6	138.7±14.4	130.6±12.1	0.005	82.2±8.2	80.1±6.6	0.227
p value**	<0.001	<0.001		<0.001	<0.001	
Effect size (95% CI)	1.2(0.76-1.63)			1.14(0.7-1.57)		
P _baseline-week2_	0.02 0.001			0.002 0.001		
P _baseline-week4_	0.001 0.001			0.001 0.001		
P _baseline-week6_	0.001 0.001			0.001 0.001		

* *t *test and ** Repeated-measures ANOVA. SBP: systolic blood pressure; DBP: diastolic blood pressure.

**Table 3:** Time effect, treatment effect, and time-treatment interaction after adjusting for baseline SBP and DBP

**Effect**	**SBP**		**DBP**	
	**F**	**p ** **value**	**F**	**p** **value**
Time	55.95	<0.001	55.56	<0.001
Treatment	1.78	0.18	0.68	0.40
Time*Treatment interaction	12.29	<0.001	11.56	<0.001

SBP: systolic blood pressure; DBP: diastolic blood pressure.

**Table 4:** Blood biochemical factors before and after 6 weeks of intervention

**Test**	**Group**	**Baseline** Mean±SD	**p** value*	**Week 6** Mean±SD	**p ** value*	**p ** value**
FBS (mg/dl)	Control	100.65±10.31	0.4	102.52±11.11	0.04	0.12
	Ma’aljobon	98.29±15.31		97.07±13.78		0.28
Cholesterol (mg/dl)	Control	180.67±42.43	0.1	180.6±42.43	0.2	0.99
	Ma’aljobon	195.28±45.40		189.82±37.44		0.15
TG (mg/dl)	Control	148.12±73.61	0.7	137.85±63.41	0.5	0.07
	Ma’aljobon	153.08±62.82		145.91±59.95		0.16
LDL (mg/dl)	Control	108.80±35.81	0.2	105.7±33.82	0.2	0.49
	Ma’aljobon	116.82±34.86		113.85±32.49		0.29
HDL (mg/dl)	Control	40.99±8.20	0.7	43.39±8.18	0.8	0.03
	Ma’aljobon	41.56±10.69		43.69±11.79		0.004
AST (U/L)	Control	21.05±5.72	0.1	21.15±7.11	0.1	0.90
	Ma’aljobon	22.82±6.86		21.84±7.60		0.10
ALT (U/L)	Control	21.68±9.71	0.01	21.27±10.15	0.1	0.74
	Ma’aljobon	28.09±13.76		24.89±13.14		0.003
HCT(%)	Control	42.53±6.15	0.1	42.03±6.40	0.5	0.09
	Ma’aljobon	44.21±5.57		42.7±5.34		0.001

^*^ *t* test (between group comparison). FBS: fasting blood sugar; TG: triglyceride; LDL, low-density lipoprotein; HDL, high-density lipoprotein; AL: alanine aminotransferase; AST: aspartate aminotransferase; HCT: hematocrit; PLT: platelet. ** Within group comparison

### Randomization and blinding

Using block randomization, the patients were divided into 2 groups. To create the randomization list, 4-block combinations (AABB, ABAB, BABA, BBAA, ABBA, and BAAB) were randomly chosen and content was unknown to both patients and researcher (double-blind). For randomization concealment, opaque sealed envelopes bearing sequential numbers were chosen.

### Statistical methods

The data were analyzed using SPSS version 17 (SPSS Inc., Chicago, IL, USA). Mean (standard deviation) was used to describe the quantitative variables, and frequency was used to describe the qualitative variables. Chi-square test was used to compare qualitative factors between the 2 groups, and a *t* test was used to examine quantitative data.

The impact of the therapy on SBP and DBP was evaluated using a repeated-measures analysis of variance (ANOVA). A Kolmogorov-Smirnov test was used to confirm that the variables were distributed normally. The sphericity was evaluated using Mauchly’s test. *P* values less than 0.05 were considered statistically significant.

## Results

**Participants demographic data**

During this study, 300 HTN patients were referred to the Birjand Cardiology Clinic, where eligibility was determined. Of the 114 patients who qualified, 57 (58.8%) were assigned to the Ma’aljobon group and 40 (41.2%) to the maltodextrin control group, for a total of 97 trial participants. Seventeen participants in the maltodextrin control group were removed from the trial due to commuting issues (4 cases), kidney stone surgery (1 case), drug taste issues (6 cases), severe HTN (BP>180/110 mmHg; 2 cases), and personal reasons (4 cases). Figure 1 displays the CONSORT (Consolidated Standards of Reporting Trials) flow diagram for the investigation.

Table 1 compares the 97 participants who completed the trial with those who dropped out by listing the demographic and baseline characteristics of the patients. There was no significant difference between the two groups in age (year), sex, marriage status, job status, smoking, duration of HTN (months), medication use, duration of medication use (months), and the type of used drugs for HTN (p>0.05). No significant disparity in the distribution of standard medications used by patients in either group was seen.

**Outcome measures**

**Primary outcome**

The mean±SD values of SBP and DBP for each group at various time intervals are presented in Table 2. After 6 weeks of treatment, both groups’ SBP and DBP significantly dropped, as shown in Table 2; with a significant decrease in the Ma’aljobon group compared to the Maltodextrin control group The trend of changes for SBP and DBP is shown in Figures 2 and 3. 

SBP and DBP significantly differ between the groups at baseline; hence, the results of repeated measures ANOVA were adjusted for baseline BP and are shown in Table 3. Based on Table 3, the time effect was statistically significant and SBP as well as DBP decreased in both groups over time. Also, regarding the type of intervention, the amount of changes in the different follow-up times was significantly different (time*treatment interaction) (p<0.001).

**Secondary outcomes**

Table 4 shows blood biochemical factors before and after the interventions in each group. At week 6, FBS (p=0.04) and PLT (p=0.03) significantly differed between the groups. No adverse effects were reported.

## Discussion

Anecdotal evidence exists about the utilization of Ma'aljobon in the treatment of “*Imtila*”, or its analogous condition, HTN. In this regard, the objective of the present study was to assess the hypotensive effect of Ma'aljobon as an adjuvant therapy in patients with uncontrolled grade I HTN. The results indicated that the administration of 50 g of Ma'aljobon per day as an adjunctive therapy exerted a more hypotensive effect (SBP: -19.7 mmHg and DBP: -13.2 mmHg) in comparison to the placebo group (p<0.001).

Corroborating these findings, Fekete et al. (2016) demonstrated that the administration of 56 g of WP per day for 8 weeks could significantly reduce 24-hr BP (SBP: -3.9 mmHg and DBP: -2.5 mmHg; p = 0.05 for both) in comparison to the control group. They ascribed the BP-lowering mechanism of WP to an augmentation of angiotensin-converting enzyme inhibitor (ACEI) activity, an ameliorated vascular endothelial state, and an improved lipid profile observed in the WP group (Fekete et al., 2016). The discrepancy in BP reduction between this study and our results may be attributed to the different whey preparation methods used in our study, derived from Persian medicine sources and validated in Mirabzadeh's study, which ensured the highest quality and efficacy of the Ma'aljobon (Mirabzadeh et al., 2015).

In another study, Yang et al. (2019) demonstrated that the daily consumption of 30 g of WP for 12 weeks led to a significant reduction in SBP (-5.1 mmHg, p=0.05) among pre- and mildly hypertensive adults who were also overweight or obese, in comparison to the placebo group. Furthermore, they observed a significant increase (p=0.04) in flow-mediated dilation (FMD) in the overweight participants of the WP group, indicating that WP can enhance endothelial function (Yang et al., 2019). However, in our study, both SBP and DBP decreased in the Ma'aljobon group compared to the control group after 6 weeks. The difference in effect and its onset may be potentially attributed to two possible factors. Firstly, the dosage in our study was higher than in Yang et al. study. Secondly, differences in the WP preparation method might also play a role.

Teunissen-Beekman et al. (2012) highlighted the potential benefits of protein supplementation in BP management among overweight individuals with upper-range prehypertension and grade 1 HTN in their study (Teunissen-Beekman et al., 2012). As previously mentioned, the quality of WP obtained from various methods is assessed by evaluating the content of its major proteins, ALA and BLG and we selected a preparation method that yielded the highest concentrations of them, based on the study by Mirabzadeh et al (Mirabzadeh et al., 2015). Heine et al. found that ALA (a primary constituent of WP) is one of the body’s richest sources of amino acids (Heine et al., 1996). It has a high nutritional value, and its biological value is higher than that of other milk proteins. It has a large amount of tryptophan (4 per molecule) and the amino acid is believed to exert its anti-hypertensive dietary potential through enhancements of central 5-hydroxytryptamine (5-HT) synthesis as well as serotonin platelet uptake (Heine et al., 1996; Panesar and Kennedy, 2012). Tryptophan amino acid, which rises after taking WP (Ma’aljobon), is a precursor to the neurotransmitters melatonin and serotonin (Markus et al., 2002). According to a Scheer et al. study, melatonin can also lower BP (Scheer et al., 2004).

Additionally, some analysis of the amino acids’ accessibility and digestibility of WP (Ma’aljobon) has demonstrated that these proteins, unlike casein, do not coagulate in the stomach’s acidic environment but instead penetrate the jejunum quickly, where their slower hydrolysis promotes the absorption (Boirie et al., 1997). For this reason, these proteins are referred to as “fast proteins” (Deponte, 2013).

Furthermore, similar to other varieties of WP, Ma'al jobon is also likely to possess ACEI properties and improves endothelial function, which, coupled with its high rate of absorption, could largely account for its more potent effect. These properties may be the reasons that could potentially justify the more pronounced effect of Ma'al jobon in comparison to placebo. However, the precise mechanism of action of Ma'al jobon supplements in the management of HTN deserves dedicated research efforts. These preliminary clinical findings may support the use of it for the management of “*Imtila*” (or HTN). 

Another finding of the present study was the significant effect of Ma'aljobon in reducing blood glucose levels (BGL) in hypertensive patients (p=0.04). A study found the blood glucose-lowering effects of WP in mitigating postprandial glucose levels (Almario et al., 2017). Another study demonstrated that hydrolysates derived from ALA in encapsulated forms have exhibited the capacity to reduce BGL and elevate insulin and glucagon-like peptide-1 (GLP-1) levels (Puri et al., 2023). These findings are congruent with our observation.

In conclusion, the significant hypotensive effect of the corn-derived placebo (maltodextrin) in our study (p<0.001) warrants further exploration. A 2019 meta-analysis of randomized controlled trials revealed that combining corn silk tea with conventional antihypertensive medications resulted in a more significant reduction in BP compared to medications alone, highlighting the potential of corn silk as a complementary approach to HTN management (Shi et al., 2019). Although our study utilized dried corn seeds, this evidence suggests that they may share antihypertensive properties with corn silk. Consequently, it is recommended to avoid using maltodextrin derived from corn in future HTN studies.

There were some limitations in this study. According to Persian medicine literature, Ma'aljobon is a liquid substance traditionally created by coagulating milk with sour acids while it is still liquid. However, as it is susceptible to rancidity in a liquid form, employing and sustaining liquid Ma'aljobon for a 6-week trial with a sizable sample number was not feasible. Thus, Ma'aljobon powder was employed in this experiment. The benefits of liquid Ma'aljobon for HTN may be greater. Future research should explore the potential advantages of liquid Ma'aljobon compared to the powdered form used in this study. 

The traditional use of Ma’aljobon as an adjuvant in managing HTN may be suitable for mild hypertensive patients. However, the study did not investigate the mechanism of Ma’aljobon. Further, larger controlled comparative clinical studies are necessary to verify its efficacy and mechanism of action. Therefore, it is recommended to incorporate Ma’aljobon as an adjuvant to antihypertensive medication under monitored consumption of both drugs.

## References

[1] Almario RU, Buchan WM, Rocke DM, Karakas SE (2017). Glucose-lowering effect of whey protein depends upon clinical characteristics of patients with type 2 diabetes. BMJ Open Diabetes Res Care.

[2] Barbiroli A, Iametti S, Bonomi F (2022). Beta-lactoglobulin as a model food protein: How to promote, prevent, and exploit its unfolding processes. Molecules.

[3] Boirie Y, Dangin M, Gachon P, Vasson MP, Maubois JL, Beaufrère B (1997). Slow and fast dietary proteins differently modulate postprandial protein accretion. Proc Natl Acad Sci.

[4] Brownlow S, Cabral JHM, Cooper R, Flower DR, Yewdall SJ, Polikarpov I, North AC, Sawyer L (1997). Bovine β-lactoglobulin at 8 Å resolution—still an enigmatic lipocalin. Structure.

[5] Cade JR, Fregly MJ, Privette M (1990). Effect of L-tryptophan on the blood pressure of patients with mild to moderate essential hypertension. Amino Acids: Chemistry, Biology and Medicine.

[6] Deponte M (2013). Glutathione catalysis and the reaction mechanisms of glutathione-dependent enzymes. Biochim Biophys Acta - Gen Subj.

[7] Fekete AA, Giromini C, Chatzidiakou Y, Givens DI, Lovegrove JA (2016). Whey protein lowers blood pressure and improves endothelial function and lipid biomarkers in adults with prehypertension and mild hypertension: results from the chronic Whey2Go randomized controlled trial. Am J Clin Nutr.

[8] Geng Sheng GS, Jiang ZhaoJing JZ, Ma HanJun MH, Wang Yu WY, Liu BenGuo LB, Liang GuiZhao LG (2020). Interaction mechanism of flavonoids and bovine β-lactoglobulin: experimental and molecular modelling studies. Food Chem.

[9] Ghods R, Gharooni M, Amin G, Nazem E, Nasrabadi AN (2014). Hypertension from the perspective of Iranian traditional medicine. Iran Red Crescent Med J.

[10] Heine W, Radke M, Wutzke K, Peters E, Kundt G (1996). α‐Lactalbumin‐enriched low‐protein infant formulas: a comparison to breast milk feeding. Acta Paediatrica.

[11] Yang T, Zubcevic J (2017). Gut-brain axis in regulation of blood pressure. Front Physiol.

[12] Hosseini A, Azadbakht M, Yousofpoor M (2017). ‘Maoljobon’A drug in Iranian Traditional Medicine. J Mazandaran Univ Med Sci.

[13] Hou E, Sun N, Zhang F, Zhao C, Liang M, Tian Z (2017). Malate and aspartate increase L-arginine and nitric oxide and attenuate hypertension. Cell Rep.

[14] Hou Y, Yin Y, Wu G (2015). Dietary essentiality of “nutritionally non-essential amino acids” for animals and humans. Exp Biol Med.

[15] Lönnerdal B (2014). Infant formula and infant nutrition: bioactive proteins of human milk and implications for composition of infant formulas. Am J Clin Nutr.

[16] Madureira A, Tavares T, Gomes A, Pintado M, Malcata FX (2010). Invited review: physiological properties of bioactive peptides obtained from whey proteins. J Dairy Sci.

[17] Madureira AR, Pereira CI, Gomes AM, Pintado ME, Malcata FX (2007). Bovine whey proteins–Overview on their main biological properties. Food Res Int.

[18] Markus CR, Olivier B, de Haan EH (2002). Whey protein rich in α-lactalbumin increases the ratio of plasma tryptophan to the sum of the other large neutral amino acids and improves cognitive performance in stress-vulnerable subjects. Am J Clin Nutr.

[19] Mathai JK, Liu Y, Stein HH (2017). Values for digestible indispensable amino acid scores (DIAAS) for some dairy and plant proteins may better describe protein quality than values calculated using the concept for protein digestibility-corrected amino acid scores (PDCAAS). Br J Nutr.

[20] Mills KT, Stefanescu A, He J (2020). The global epidemiology of hypertension. Nat Rev Nephrol.

[21] Mirabzadeh M, Khanavi M, Golestani A, Shams-ardekani MR, Rahimi R, Sahraei Z, Moghaddam G, Khoshayand MR, Hajimahmoodi M (2015). Effects of heating and acidic solutions of vinegar and oxymel on milk coagulation for identification and quantification of resulting α-lactalbumin and β-lactoglobulin proteins in the final whey product. Anal Chem Lett.

[22] Miura K, Torii S (2012). Diet, nutrients, and the prevention of hypertension. Curr Nutr Rep.

[23] Navabzadeh M, Hashem-Dabaghian F, Kazemi T, Shojaii A, Nakhaei I, Hadinia J, Ghods R ((2019a)). Effect of a Persian medicine preparation, Ma'aljobon, on constipation in patients with hypertension. J Res Med Sci.

[24] Navabzadeh M, Hashem-Dabaghian F, Shojaii A, Kazemi T, Hadinia J, Ghods T, Ghods R (2019b). The effect of a kind of whey protein (Ma'oljobon) on Insomnia: A randomized clinical trial. Complement Ther Clin Pract.

[25] Panesar PS, Kennedy JF (2012). Biotechnological approaches for the value addition of whey. Crit Rev Biotechnol.

[26] Pasalar M (2021). Persian medicine as a holistic therapeutic approach. Curr Drug Discov Technol.

[27] Puri B, Meena S, Kumar MH S, Shelke PA, Sabikhi L (2023). Encapsulation and assessment of antidiabetic potential of α-Lactalbumin-derived hydrolysates. J Agric Food Chem.

[28] Rebholz CM, Friedman E, Powers LJ (2012). Dietary protein intake and blood pressure: a meta-analysis of randomized controlled trials. Am J Epidemiol.

[29] Sandström O, Lönnerdal B, Graverholt G, Hernell O (2008). Effects of α-lactalbumin–enriched formula containing different concentrations of glycomacropeptide on infant nutrition. Am J Clin Nutr.

[30] Scheer FA, Van Montfrans GA, van Someren EJ, Mairuhu G, Buijs RM (2004). Daily nighttime melatonin reduces blood pressure in male patients with essential hypertension. Hypertension.

[31] Shi S, Li S, Li W, Xu H (2019). Corn silk tea for hypertension: a systematic review and meta-analysis of randomized controlled trials. J Evid Based Complementary Altern Med.

[32] Siahpoosh MB (2012). Six essential principles of Iranian traditional medicine for maintaining health from the Quran’s point of view. Quran and Medicine.

[33] Teunissen-Beekman KF, Dopheide J, Geleijnse JM, Bakker SJ, Brink EJ, de Leeuw PW, van Baak MA (2012). Protein supplementation lowers blood pressure in overweight adults: effect of dietary proteins on blood pressure (PROPRES), a randomized trial. Am J Clin Nutr.

[34] Tian Z, Liang M (2021). Renal metabolism and hypertension. Nat Commun.

[35] Williams B, Mancia G, Spiering W, Agabiti Rosei E, Azizi M, Burnier M, Clement DL, Coca A, de Simone G, Dominiczak A, Kahan T, Mahfoud F, Redon J, Ruilope L, Zanchetti A, Kerins M, Kjeldsen SE, Kreutz R, Laurent S, Lip GYH, McManus R, Narkiewicz K, Ruschitzka F, Schmieder RE, Shlyakhto E, Tsioufis C, Aboyans V, Desormais I (2018). 2018 ESC/ESH Guidelines for the management of arterial hypertension: The Task Force for the management of arterial hypertension of the European Society of Cardiology (ESC) and the European Society of Hypertension (ESH). Eur Heart J.

[36] Yang J, Wang HP, Tong X, Li ZN, Xu JY, Zhou L, Zhou BY, Qin LQ (2019). Effect of whey protein on blood pressure in pre‐and mildly hypertensive adults: A randomized controlled study. Food Sci Nutr.

[37] Yang P, Zhou L, Chen M, Zeng L, Ouyang Y, Zheng X, Chen X, Yang Z, Tian Z (2022). Supplementation of amino acids and organic acids prevents the increase in blood pressure induced by high salt in Dahl salt-sensitive rats. Food Funct.

